# Association Between Socioeconomic Status and Asthma-Related Emergency Department Visits Among World Trade Center Rescue and Recovery Workers and Survivors

**DOI:** 10.1001/jamanetworkopen.2020.1600

**Published:** 2020-03-23

**Authors:** Jennifer Brite, Howard E. Alper, Stephen Friedman, Erin Takemoto, James Cone

**Affiliations:** 1 Division of Epidemiology, New York City Department of Health and Mental Hygiene, World Trade Center Health Registry, New York, New York

## Abstract

**Importance:**

Although the association between poor economic or social standing and health is well established, few studies have attempted to examine the mediational pathways that produce adverse outcomes in disadvantaged populations.

**Objective:**

To determine whether barriers to care mediate the association between socioeconomic status (SES) and asthma-related emergency department (ED) visits.

**Design, Setting, and Participants:**

This cohort study used data from the World Trade Center Health Registry, which comprises rescue and recovery workers and community members who worked, lived, studied or were otherwise present in downtown Manhattan, New York, during or immediately after the September 11, 2001, disaster. Data were matched to an administrative database of ED visits. Those who experienced an asthma-related ED visit and those who did not were compared in bivariate analysis. A mediation analysis was conducted to determine the role of barriers to care in the association between number of ED visits and SES.

**Exposures:**

Education, income, and race/ethnicity, which were collected at first survey in 2003 to 2004.

**Main Outcomes and Measures:**

Asthma-related ED visits that occurred after survey responses regarding barriers to care were collected (2006-2007) but before 2016, the latest date that data were available.

**Results:**

The analytic sample included 30 452 enrollees (18 585 [61%] male; median [interquartile range] age, 42.0 [35.0-50.0] years; 20 180 [66%] white, 3834 [13%] African American, and 3961 [13%] Hispanic or Latino [any race]). Approximately half (49%) had less than a bachelor’s degree, and 15% had an annual income less than $35 000. Those of lower SES were more likely to experience an asthma-related ED visit. Although number of barriers to care mediated this association, they explained only a small percentage of the overall health disparity (ranging from 3.0% [95% CI, 2.3%-3.9%]) of the differences between African American and white individuals to 9.8% [95% CI, 7.7%-11.9%]) comparing those with less than a high school diploma to those with at least a bachelor’s degree. However, the association varied by specific barrier to care. Lack of money, insurance, and transportation mediated up to 11.8% (95% CI, 8.1%-15.9%), 12.5% (95% CI, 8.5%-17.4%), and 4.3% (95% CI, 1.7%-8.4%), respectively, of the association between SES and number of ED visits. Lack of childcare, not knowing where to go for care, and inability to find a health care professional mediated a smaller or no percentage of the association.

**Conclusions and Relevance:**

The identification of vulnerable subpopulations is an important goal to reduce the burden of asthma-related hospital care. More research is needed to fully understand all of the pathways that lead disaster survivors of lower SES to disproportionately experience ED visits due to asthma.

## Key Points

**Question:**

How much of the association between socioeconomic status and asthma-related emergency department visits is mediated by barriers to care?

**Findings:**

In this cohort study of 30 452 enrollees in the World Trade Center Health Registry, lower socioeconomic status was associated with a greater number of asthma-related emergency department visits, and barriers to care mediated a small percentage of this association.

**Meaning:**

These findings suggest that individuals of lower socioeconomic status in the World Trade Center Health Registry experience worse asthma outcomes despite being potentially eligible for no-cost treatment and medications.

## Teaser

This cohort study explores the association between socioeconomic status and asthma-related visits to the emergency department (ED) among individuals enrolled in the World Trade Center Health Registry.

## Introduction

The association between health outcomes and income, education, race/ethnicity, and other proxies of socioeconomic status (SES) has been well described, but the causal drivers of this association remain poorly understood. The precise pathways that lead those of lower SES to have poorer health outcomes compared with those of higher SES remain surprisingly understudied owing to lack of large-scale studies with information appropriate for mediational analysis.

Asthma-related emergency department (ED) visits are strongly associated with SES in the general patient population^1,2,3^ and may be an indicator of poorly controlled asthma due to factors such as inability to access primary care or lack of response to treatment. A similar pattern of asthma-related health disparities exists in the World Trade Center Health Registry exposed cohort,^4^ despite the fact that the World Trade Center Health Program (WTCHP) provides eligible participants with treatment and medications at no out-of-pocket cost for asthma related to the September 11, 2001, disaster (9/11).

It is not known whether barriers to care, such as delays in WTCHP participation, long waiting times for appointments, and lack of coverage for comorbid conditions, differentially affect those with fewer resources. It is also unclear whether experiencing these barriers could, in turn, lead patients to resort to obtaining acute care through EDs. In other words, although treatment for asthma is available to all qualifying rescue and recovery workers and community members, those of lower SES may not be able to access or use it as effectively as those who have higher SES.

The World Trade Center Health Registry provides a novel data set to answer these questions because it has a rich set of longitudinal data that may shed light on why health disparities persist even in populations that are, for the most part, eligible to receive the same or very similar care. Previous research^5,6,7,8^ has shown that universal health care ameliorates but does not eliminate inequality because it may not address all contributing structural factors. Even under universal coverage, patients may not receive equivalent care in a complex health care system. The WTCHP provides a unique perspective because for those enrolled, specialized clinics for 9/11-related illness offer standardized care with highly specific treatment protocols.

The aim of the present study is to determine whether various measures of SES are associated with acute care and whether the association is mediated by barriers to care. This analysis builds on previous work on this topic by using additional data through 2016 and also formally testing whether barriers to care mediate the association between SES and asthma hospitalizations.^4^ It is also the first study, to our knowledge, to explicitly examine these pathways in a disaster-exposed population with elevated rates of respiratory illness.^9^

## Methods

### Study Population

The World Trade Center Health Registry comprises 71 426 enrollees who lived, worked, or were passing through lower Manhattan, New York, on September 11, 2001, or who conducted rescue and recovery work in the months that followed. The prospective cohort is made up of 4 waves spanning 2003 to 2016, in which enrollees self-reported both their exposure to the terrorist attack as well as their health conditions before and after 9/11.^9^ The registry protocol was approved by the Centers for Disease Control and Prevention and New York City Department of Health and Mental Hygiene institutional review boards. This approval applied to the current study as well. All enrollees provided verbal informed consent. This study followed the Strengthening the Reporting of Observational Studies in Epidemiology (STROBE) reporting guideline.

### Analytic Study Sample

Because educational attainment is generally not completed until age 25 years and early-life income may not be indicative of adult income, the current study excluded the 7780 enrollees younger than 25 years on 9/11. We also removed the 20 038 enrollees who did not participate in the second survey when barriers to care were collected and 4786 who were missing any exposure or covariate data. We further excluded 8370 enrollees who did not reside in New York State because hospital admission data were only available from that state. Our final analytic sample comprised 30 452 enrollees. To capture hospitalizations among those without an asthma diagnosis and also to address the possibility that those of low SES may differentially report asthma status, the sample included those who self-reported asthma and those who reported no asthma. We included both those who self-reported first diagnoses before and after 9/11. However, in a sensitivity analysis, we restricted the sample to those who self-reported post-9/11 asthma at baseline or at wave 2.

### Exposure: SES

Our exposure of interest, SES, was defined in 2 distinct ways: income, and educational attainment. We also examined race/ethnicity, as it is associated with poor health owing to structural racism. All 3 exposures were self-reported at baseline (wave 1). For race/ethnicity, those who reported Hispanic or Latino of any race were considered Latino and the remaining enrollees were categorized as white, African American, Asian, or other. Total household income in 2002 was collapsed into 4 categories: less than $35 000, $35 000 to less than $75 000, $75 000 to less than $200 000, and $200 000 or greater. Education was reported as highest grade or year of school completed and categorized as less than high school, high school or General Educational Development only, some college, or bachelor’s degree or higher.

### Covariates

Demographic covariates, age at 9/11 and sex, were measured at wave 1. We included these demographic characteristics because they are associated with both SES and asthma exacerbation, which may lead to greater number of ED visits.

### Mediators: Barriers to Care

Barriers to care were collected in the second World Trade Center Health Registry survey in the years of 2006 and 2007. Barriers to care questions were asked in the context of an enrollee’s general health care experience in the last 12 months and not specifically about the WTCHP. Respondents were asked to check all that applied for the following barriers to care: lacked money, lacked insurance, lacked transportation, lacked childcare, did not know where to go for care, was unable to find a health care professional who could diagnose my condition, and other (please specify).

First, we summed the number of barriers to care per person. Next, we conducted separate analyses for each type of barrier of care. Owing to a great heterogeneity of responses, we did not subcategorize the free-text answers provided under other but instead included them as 1 barrier to care if an enrollee answered affirmatively when barriers to care were summed.

### Outcome: Asthma-Related ED Visits

Registry data were matched via a linkage to the New York State Department of Health’s Statewide Planning and Research Cooperative System database, which is made up of all hospital and ED discharges in New York except those that occur in federal and psychiatric hospitals. Records were matched based on an algorithm using parts of the first and last names, as well as date of birth, sex, Social Security number, and zip code in hierarchical rounds. Asthma-related hospital ED visits were determined via *International Classification of Diseases, Ninth Revision, Clinical Modification (ICD-9-CM)* and *International Statistical Classification of Diseases, Tenth Revision, Clinical Modification (ICD-10-CM)* codes as a principal diagnosis or a respiratory condition listed as the principal diagnosis and asthma listed as a secondary diagnosis. For this analysis, the number of ED visits were summed for each enrollee. To establish temporality, we ensured that SES measures were collected at baseline (2003-2004), barriers to care were collected in the second survey (2006-2007), and all asthma ED visits took place after respondents answered the second survey (2007-2016). In a sensitivity analysis, we created 2 additional outcomes: the first summed inpatient asthma stays (a proxy for disease severity) and the second summed both ED and inpatient asthma stays.

### Statistical Analysis

Bivariate analyses were conducted using the χ^2^ test to compare the distribution of sociodemographic factors between those who visited an ED for asthma and those who did not. Next, we calculated predicted ED visits rates per 100 enrollees via Poisson regression separately for race/ethnicity (with race/ethnicity, age, and sex as offsets), income (with income, age, and sex as offsets), and education (with education, age, and sex as offsets).

Additionally, we conducted an analysis to determine whether barriers to care mediated the association between SES and asthma ED visits. The proposed mediational pathways are illustrated in the Figure. We used mediation analysis as described by Imai et al.^10,11^ In brief, this approach calculates average mediation and direct effect sizes by simulating predicted potential values, which cannot be observed, of the outcome or mediator and then decomposing those effect sizes into various quantities of interest (direct, indirect, and total effect sizes). The general steps of this process are described by Hicks et al.^12^ Because mediators were highly correlated with one another, we created separate statistical models for each. This analysis was conducted using the R mediation package (R Project for Statistical Computing). We calculated total, direct, and indirect effect sizes as well as percentage mediated = indirect effect / (direct effect + indirect effect) × 100.

**Figure.**
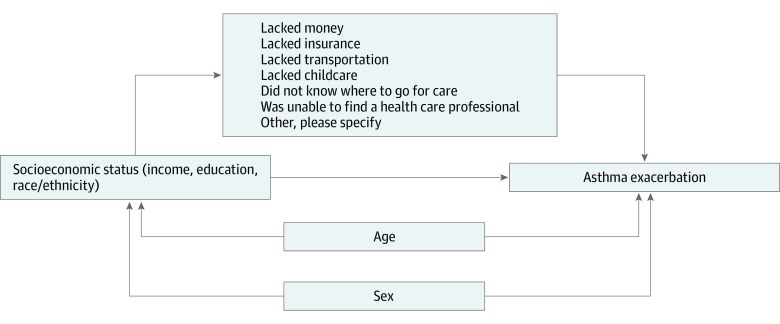
Proposed Mediational Pathways

The threshold for statistical significance was set at 2-sided *P* < .05. All analyses were conducted in SAS version 9.4 (SAS Institute) and RStudio version 1.0.143.

## Results

The analytic sample comprised 30 452 enrollees (18 585 [61%] male; median [interquartile range] age, 42.0 [35.0-50.0] years; 20 180 [66%] white, 3834 [13%] African American, and 3961 [13%] Hispanic or Latino [any race]). Approximately half (49%) had less than a bachelor’s degree, and 50% had an income of more than $75 000 annually, with 15% having an annual income less than $35 000. Among those with at least 1 ED visit, the median (IQR) number of visits for the entire period was 1 (1-2). Four hundred forty-eight enrollees had at least 1 ED visit during the study period. Twenty percent of the sample had at least 1 barrier to care, and having a barrier to care was associated with having at least 1 asthma-related ED visit. Twenty-eight percent of those with at least 1 ED had at least 1 barrier to care, while only 19% of those with no hospitalizations did (*P* < .001). Race, sex, income, and educational attainment were associated with having at least 1 ED visit in bivariate analyses (Table 1). White and Asian individuals, those with more education, and those with higher income had lower rates of asthma ED visits compared with other racial/ethnic groups and those of lower SES (Table 2, Table 3, and Table 4). Barriers to care were more common among those with nonwhite race/ethnicity and those with the lowest levels of income and education (eTable 1 in the Supplement).

**Table 1.** Study Characteristics by Any Asthma-Related ED Visit

Characteristic	No. (%)			*P* value^a^
	Overall (N = 30 452)	ED visit (n = 448)	No ED visit (n = 30 004)	
Exposure				
Race				
White	20 180 (66)	157 (35)	20 023 (67)	<.001
Black or African American	3834 (13)	138 (31)	3696 (12)	
Hispanic or Latino, any race	3961 (13)	129 (29)	3832 (13)	
Asian, including Native Hawaiian/Pacific Islander	1697 (6)	7 (2)	1690 (6)	
Multiracial or other	780 (3)	17 (4)	763 (3)	
Education				
Less than high school	1276 (4)	35 (8)	1241 (4)	<.001
High school only	5914 (19)	123 (27)	5791 (19)	
Some college	7679 (25)	165 (37)	7514 (25)	
At least a bachelor's degree	15 583 (51)	125 (28)	15 458 (52)	
Income, $				
<35 000	4537 (15)	155 (35)	4382 (15)	<.001
35 000 to <75 000	10 691 (35)	178 (40)	10 513 (35)	
75 000 to <200 000	13 139 (43)	108 (24)	13 031 (43)	
≥200 000	2085 (7)	7 (2)	2078 (7)	
Mediator				
Any barrier to care				
No	24 485 (80)	322 (72)	24 163 (81)	<.001
Yes	5967 (20)	126 (28)	5841 (19)	
Lack of money				
No	28 579 (94)	392 (88)	28 187 (94)	<.001
Yes	1873 (6)	56 (13)	1817 (6)	
Lack of insurance				
No	28 852 (95)	400 (89)	28 452 (95)	<.001
Yes	1600 (5)	48 (11)	1552 (5)	
Lack of transportation				
No	30 221 (99)	438 (98)	29 783 (99)	<.001
Yes	231 (1)	10 (2)	221 (1)	
Lack of childcare				
No	30 194 (99)	445 (99)	29 749 (99)	.68
Yes	258 (1)	3 (1)	255 (1)	
Did not know where to go for care				
No	29 686 (97)	433 (97)	29 253 (97)	.26
Yes	766 (3)	15 (3)	751 (3)	
Unable to find health care professional				
No	29 715 (98)	429 (96)	29 286 (98)	.01
Yes	737 (2)	19 (4)	718 (2)	
Covariate				
Sex				
Male	18 585 (61)	202 (45)	18 383 (61)	<.001
Female	11 867 (39)	246 (55)	11 621 (39)	
Age, median (IQR), y	42.0 (35.0-50.0)	40.0 (35.0-47.0)	42.0 (35.0-50.0)	.01
Self-reported asthma				
Yes	5897 (19)	342 (77)	5555 (19)	<.001
No	24 330 (80)	101 (23)	24 229 (81)	
Do not know	19 (0)	0	19 (0)	
Refused	1 (0)	0	1 (0)	

Abbreviations: ED, emergency department; IQR, interquartile range.

^a^ *P* value comparisons across treatment groups for categorical variables are based on χ^2^ test of homogeneity; *P* values for continuous variables are based on analysis of variance or Kruskal-Wallis test for median.

**Table 2.** Direct and Indirect Effect Sizes of Race on Number of Asthma-Related ED Visits^a^

Race or mediator	ED visits per 100 enrollees, difference in No. (95% CI)	*P* value	Mediated, % (95% CI)^b^
**Category**			
White	1.1 (0.9 to 1.3)^c^		
African American	12.4 (11.2 to 13.8)^c^		
Hispanic	5.9 (5.1 to 6.8)^c^		
Asian	0.7 (0.4 to 1.2)^c^		
Other	3.8 (2.7 to 5.4)^c^		
**African American vs white**			
Total effect	12.5 (11.3 to 13.7)^d^	<.001	
Indirect effects			
Lack of money	0.5 (0.4 to 0.7)^d^	<.001	3.9 (2.7 to 5.2)
Lack insurance	0.5 (0.3 to 0.7)^d^	<.001	3.8 (2.6 to 5.4)
Lack transportation	0.5 (0.3 to 0.7)^d^	<.001	3.5 (2.2 to 5.2)
Lack of childcare	0.0 (0.0 to 0.0)^d^	.56	0.0 (−0.1 to 0.1)
Did not know where to go for care	0.1 (0.0 to 0.3)^d^	.04	1.0 (0.0 to 2.1)
Unable to find a health care professional	0.0 (0.0 to 0.0)^d^	.78	0.0 (−0.3 to 0.3)
No. of barriers	0.4 (0.3 to 0.5)^d^	<.001	3.0 (2.3 to 3.9)
**Hispanic vs white**			
Total effect	5.3 (4.6 to 6.1)^d^	<.001	
Indirect effects			
Lack of money	0.4 (0.3 to 0.5)^d^	<.001	6.6 (5.0 to 8.5)
Lack insurance	0.4 (0.3 to 0.5)^d^	<.001	6.6 (5.0 to 8.7)
Lack transportation	0.2 (0.1 to 0.3)^d^	<.001	3.8 (2.5 to 5.7)
Lack of childcare	0.0 (0.0 to 0.0)^d^	.53	0.0 (−0.2 to 0.1)
Did not know where to go for care	0.2 (0.1 to 0.3)^d^	<.001	3.6 (2.4 to 5.5)
Unable to find a health care professional	0.0 (0.0 to 0.0)^d^	.80	−0.1 (−0.5 to 0.5)
No. of barriers	0.4 (0.3 to 0.4)^d^	<.001	6.6 (5.5 to 8.1)
**Asian vs white**^**e**^			
Total effect	−0.4 (−0.8 to 0.1)^d^	.08	
Indirect effects			
Lack of money	0.1 (0.0 to 0.1)^d^	<.001	
Lack insurance	0.1 (0.0 to 0.1)^d^	<.001	
Lack transportation	0.0 (0.0 to 0.1)^d^	.01	
Lack of childcare	0.0 (0.0 to 0.0)^d^	.57	
Did not know where to go for care	0.0 (0.0 to 0.1)^d^	<.001	
Unable to find a health care professional	0.0 (0.0 to 0.0)^d^	.82	
No. of barriers	0.1 (0.0 to 0.1)^d^	<.001	
**Other vs white**			
Total effect	2.9 (1.7 to 4.5)^d^	<.001	
Indirect effects			
Lack of money	0.4 (0.2 to 0.6)^d^	<.001	11.8 (7.9 to 17.2)
Lack insurance	0.4 (0.2 to 0.6)^d^	<.001	11.9 (7.8 to 18.0)
Lack transportation	0.3 (0.1 to 0.5)^d^	<.001	9.0 (4.8 to 15.3)
Lack of childcare	0.0 (0.0 to 0.0)^d^	.57	−0.1 (−0.6 to 0.2)
Did not know where to go for care	0.1 (0.0 to 0.2)^d^	.14	1.8 (−0.9 to 5.0)
Unable to find a health care professional	0.0 (0.0 to 0.0)^d^	.85	0.0 (−0.5 to 0.4)
No. of barriers	0.2 (0.2 to 0.4)^d^	<.001	9.6 (6.7 to 12.6)

Abbreviation: ED, emergency department.

^a^ All estimates adjusted by age and sex.

^b^ Percentage mediated = indirect effect/(direct effect + indirect effect) × 100.

^c^ These values are number of visits per 100.

^d^ These values are difference in number of visits per 100.

^e^ Percentage mediated was not calculated for Asian individuals because they had lower rates of hospitalization than white individuals.

**Table 3.** Direct and Indirect Effect Sizes of Income on Number of Asthma-Related ED Visits^a^

Income level or mediator	ED visits per 100 enrollees, difference in No. (95% CI)	*P* value	Mediated, % (95% CI)^b^
**Category, $**			
<35 000	10.2 (9.2 to 11.3)^c^		
35 000 to <75 000	3.0 (2.6 to 3.4)^c^		
75 000 to <200 000	1.1 (0.9 to 1.3)^c^		
≥200 000	0.3 (0.2 to 0.6)^c^		
**<$35 000 vs ≥$200 000**			
Total effect	11.7 (10.6 to 12.7)^d^	<.001	
Indirect effects			
Lack of money	1.1 (0.9 to 1.4)^d^	<.001	9.1 (7.0 to 11.2)
Lack insurance	1.1 (0.9 to 1.4)^d^	<.001	9.0 (7.2 to 11.2)
Lack transportation	0.7 (0.5 to 0.9)^d^	<.001	
Lack of childcare	−0.1 (−0.2 to 0.1)^d^	.30	−0.6 (−1.3 to 0.5)
Did not know where to go for care	0.5 (0.4 to 0.7)^d^	<.001	4.3 (3.0 to 5.9)
Unable to find a health care professional	−0.1 (−0.1 to 0.0)^d^	.21	−0.5 (−1.1 to 0.3)
No. of barriers	1.1 (1.0 to 1.3)^d^	<.001	9.7 (8.2 to 11.6)
**$35 000 to <$75 000 vs ≥$200 000**			
Total effect	3.2 (2.7 to 3.7)^d^	<.001	
Indirect effects			
Lack of money	0.1 (0.1 to 0.2)^d^	<.001	4.4 (3.2 to 6.1)
Lack insurance	0.1 (0.1 to 0.2)^d^	<.001	3.9 (2.8 to 5.6)
Lack transportation	0.0 (0.0 to 0.1)^d^	.01	1.1 (0.4 to 1.8)
Lack of childcare	0.0 (0.0 to 0.0)^d^	.38	0.0 (−0.2 to 0.1)
Did not know where to go for care	0.1 (0.0 to 0.1)^d^	<.001	2.2 (1.4 to 3.3)
Unable to find a health care professional	0.0 (0.0 to 0.0)^d^	.20	−0.2 (−0.6 to 0.1)
No. of barriers	0.2 (0.1 to 0.2)^d^	<.001	4.8 (3.8 to 6.4)
**$75 000 to <$200 000 vs ≥$200 000**			
Total effect	0.6 (0.3 to 0.8)^d^	<.001	
Indirect effects			
Lack of money	0.0 (0.0 to 0.0)^d^	<.001	1.9 (1.0 to 4.5)
Lack insurance	0.0 (0.0 to 0.0)^d^	.01	1.4 (0.5 to 3.5)
Lack transportation	0.0 (0.0 to 0.0)^d^	.47	0.2 (−0.7 to 0.9)
Lack of childcare	0.0 (0.0 to 0.0)^d^	.27	−0.1 (−0.6 to 0.1)
Did not know where to go for care	0.0 (0.0 to 0.0)^d^	<.001	1.8 (0.8 to 4.3)
Unable to find a health care professional	0.0 (0.0 to 0.0)^d^	.23	−0.2 (−0.6 to 0.1)
No. of barriers	0.0 (0.0 to 0.0)^d^	<.001	3.5 (2.3 to 7.5)

Abbreviation: ED, emergency department.

^a^ All estimates adjusted by age and sex.

^b^ Percentage mediated = indirect effect/(direct effect + indirect effect) × 100.

^c^ These values are number of visits per 100.

^d^ These values are difference in number of visits per 100.

**Table 4.** Direct and Indirect Effect Sizes of Education on Number of Asthma-Related ED Visits^a^

Education level or mediator	ED visits per 100 enrollees, difference in No. (95% CI)	*P* value	Mediated, % (95% CI)^b^
**Category**			
At least a bachelor's degree	1.2 (1.1 to 1.4)^c^		
Some college	5.2 (4.6 to 5.9)^c^		
High school	4.9 (3.4 to 4.4)^c^		
Less than high school	7.7 (6.4 to 9.4)^c^		
**Some college vs at least a bachelor’s degree**			
Total effect	4.8 (4.2 to 5.4)^d^	<.001	
Indirect effects			
Lack of money	0.2 (0.1 to 0.3)^d^	<.001	4.5 (2.7 to 6.6)
Lack insurance	0.2 (0.1 to 0.3)^d^	<.001	3.1 (1.3 to 5.1)
Lack transportation	0.2 (0.1 to 0.4)^d^	<.001	4.7 (2.8 to 7.3)
Lack of childcare	0.0 (0.0 to 0.0)^d^	.12	−0.1 (−0.4 to 0.1)
Did not know where to go for care	0.1 (0.1 to 0.2)^d^	<.001	2.6 (1.5 to 4.1)
Unable to find a health care professional	0.0 (0.0 to 0.0)^d^	.97	0.0 (−0.2 to 0.3)
No. of barriers	0.2 (0.2 to 0.3)^d^	<.001	4.4 (3.4 to 5.5)
**High school vs at least a bachelor's degree**			
Total effect	3.2 (2.6 to 3.8)^d^	<.001	
Indirect effects			
Lack of money	0.3 (0.2 to 0.4)^d^	<.001	7.3 (4.6 to 10.8)
Lack insurance	0.2 (0.2 to 0.4)^d^	<.001	7.1 (4.5 to 10.1)
Lack transportation	0.1 (0.1 to 0.2)^d^	<.001	3.8 (1.8 to 6.4)
Lack of childcare	0.0 (0.0 to 0.0)^d^	.12	−0.3 (−0.7 to 0.1)
Did not know where to go for care	0.1 (0.1 to 0.2)^d^	<.001	3.2 (1.7 to 5.3)
Unable to find a health care professional	0.0 (0.0 to 0.0)^d^	.91	0.0 (−0.3 to 0.4)
No. of barriers	0.2 (0.1 to 0.2)^d^	<.001	5.8 (4.4 to 7.5)
**Less than high school vs at least a bachelor's degree**			
Total effect	7.5 (5.9 to 9.4)^d^	<.001	
Indirect effects			
Lack of money	1.1 (0.7 to 1.6)^d^	<.001	11.8 (8.1 to 15.9)
Lack insurance	1.1 (0.7 to 1.7)^d^	<.001	12.5 (8.5 to 17.4)
Lack transportation	0.4 (0.1 to 0.7)^d^	<.001	4.3 (1.7 to 8.4)
Lack of childcare	0.0 (0.0 to 0.0)^d^	.21	−0.2 (−0.6 to 0.1)
Did not know where to go for care	0.6 (0.4 to 1.0)^d^	<.001	7.1 (4.5 to 10.7)
Unable to find a health care professional	0.0 (−0.1 to 0.1)^d^	.95	0.0 (−0.8 to 1.0)
No. of barriers	0.7 (0.5 to 1.0)^d^	<.001	9.8 (7.7 to 11.9)

Abbreviation: ED, emergency department.

^a^ All estimates adjusted by age and sex.

^b^ Percentage mediated = indirect effect/(direct effect + indirect effect) × 100.

^c^ These values are number of visits per 100.

^d^ These values are number in number of visits per 100.

The number of barriers to care mediated the association between race and number of asthma-related ED visits. For example, African American individuals experienced more hospitalizations compared with white individuals, but lack of money, insurance, and transportation each explained only 0.5 ED visit (3.0% [95% CI, 2.3%-3.9%] of the total effect) in the difference between African American and white participants. However, some specific barriers had larger mediational effect sizes on the difference in ED visits between those who were white and those who were of other race. For example, lack of money mediated 11.8% (95% CI, 7.9%-17.2%) of the difference; lack of insurance, 11.9% (95% CI, 7.8%-18.0%); and lack of transportation, 9.0% (95% CI, 4.8%-15.3%) (Table 2).

A similar pattern was found for income. Although the effect size of number of barriers to care was relatively modest (with percentage mediated ranging from 0% to 9.7%), lack of money (9.1% [95% CI, 7.0%-11.2%]), lack of insurance (9.0% [95% CI, 7.2%-11.2%]), and lack of transportation (5.5% [95% CI, 4.0%-7.4%]) mediated some of the disparity in ED visits between those with incomes less than $35 000 and those who earned $200 000 or more. However, in absolute terms, the effect size was not large. For example, lack of money only explained 1.1 (95% CI, 0.9-1.4) additional ED visit between the lowest and highest income levels (Table 3).

Among the least and most educated, number of barriers to care only explained 0.7 ED visit (95% CI, 0.5-1.0 visit) or 9.8% (95% CI, 7.7%-11.9%) of the association. However, here again, lack of money (11.8% [95% CI, 8.1%-15.9%]), insurance (12.5% [95% CI, 8.5%-17.4%]), and transportation (4.3% [95% CI, 1.7%-8.4%]) were the strongest mediators of the association between educational attainment and ED visits (Table 4).

The results from the sensitivity analyses (1) using a sum of inpatient stays and ED visits as the outcome and (2) using a sum of inpatient stays only as an outcome were qualitatively similar to the results presented here (eTable 2 through eTable 7 in the Supplement). In the sensitivity analysis restricted only to patients who self-reported asthma, rates of ED visits were higher for all SES groups, and consequently many estimates of the indirect effect sizes were larger as well. However, the mediating effect size of barriers to care (measured by percentage mediated) appeared to be very similar to that found in the full analytic sample (eTable 8 through eTable 10 in the Supplement).

## Discussion

This study produced 2 substantive findings. First, these data demonstrate that health disparities exist in ED visit rates in the 9/11-exposed population. Second, although this association is partially mediated by barriers to care, the barriers studied here accounted for only a relatively small percentage of the total effect sizes.

The present analysis builds on the findings that race/ethnicity and education were associated with number of asthma-related inpatient stays among persons exposed to the 9/11 disaster.^4^ These differences may be driven by a few factors: increased asthma incidence, increased asthma severity, decreased asthma control, or a combination of all 3 in those with lower SES. This study’s findings are also in keeping with a previous analysis of unmet mental health needs in this cohort, which found that barriers to care were common, particularly those related to financial factors.^13^ However, this is the first study, to our knowledge, to explicitly test the role of barriers to care in SES health disparities in asthma ED visits in the World Trade Center Health Registry cohort.

Many registry enrollees are potentially eligible for health care at no out-of-pocket cost for 9/11-related conditions, such as asthma, through the federal WTCHP, and there is some evidence that those with lower income may actually apply at higher rates.^14^ However, qualitative data show that even among those covered, barriers to treatment exist that may have greater impact on those with low SES.^14^ There are a few ways in which health disparities may be exacerbated when designing a postdisaster health program. First, not all eligible patients will enroll in a program, particularly if requirements are time-consuming or unclear. After enrollment, those with lower SES may not be able to access care as readily as their counterparts with greater resources. This analysis cannot determine at what point disparities may arise, and this study is not an evaluation of quality of care at the WTCHP.

Although this study found statistically significant mediation of differences in ED visits, our analysis suggests that overall factors beyond the types of barriers to care examined primarily drive the association between SES and asthma ED visits in this cohort. These factors may include lower health literacy and greater gaps in asthma knowledge,^15,16,17^ lower self-efficacy and confidence,^15,18^ or difficulty booking or finding time for an appointment, which may be more of a burden for those with low-paying jobs with little or no paid leave. Finally, it is possible that patient characteristics or environmental hazards, such as exposure to tobacco smoke, may play a larger role than barriers to care. Many comorbidities associated with poor asthma control, such as obesity, are more common in those with low SES,^19,20^ and individuals with low SES are also much more likely to live in poor-quality housing and in neighborhoods more exposed to air pollution, which is associated with asthma triggers.^21^

Although overall number of barriers to care did not appear to play a large mediating role, it should be noted that the association varied by some individual types of barriers to care. For example, monetary factors, such as lack of insurance or money, mediated a larger percentage of the association than other barriers. Although the WTCHP covers all out-of-pocket care for 9/11-related illness, including asthma, it does not reimburse other costs, such as those related to conditions not attributable to 9/11. It is possible that although the program adequately meets the economic needs of patients with asthma who have high SES, those with low SES may have additional expenses. For example, those with more resources are able to obtain health insurance to help manage chronic conditions such as allergies, which are associated with asthma exacerbation.^22,23^ Insufficient insurance or money may also be a proxy for other barriers, such as poor or no employment. Lack of transportation was also a statistically significant mediator of the association between SES and asthma ED visits. World Trade Center Health Program Clinical Centers of Excellence, which serve the 9/11 community, are located throughout the New York metropolitan area. Although a large proportion of patients visit clinics that are located in Manhattan, which has an extensive public transportation network, some program clinicians and clinics in other areas of New York State may be located in areas with insufficient or no low-cost transportation options. Not knowing where to go to find care mediated a small proportion of the association, and being unable to find a health care professional was not a significant mediator. This suggests that this sample of 9/11-exposed individuals has been relatively well served by WTCHP communications and referrals, including the extensive outreach conducted by the World Trade Center Health Registry to refer enrollees to the WTCHP.^24^ Finally, childcare concerns did not appear to be a significant barrier to care in this cohort, which is not surprising given that this population is older, with a median age of 47 years at wave 2.

### Strengths and Limitations

This study had several strengths that should be noted. First, longitudinal data were collected for more than a decade from a large cohort of both rescue and recovery workers and community members. Next, these data was linked to administrative hospital ED visits, reducing the risk of recall bias.

However, a few limitations should also be considered. Loss to follow-up between waves 1 and 2 of the survey was greater among those of lower SES. However, it is likely this loss to follow-up is most common in those with the outcome (asthma-related ED visits), suggesting that the true association may be even greater than the estimates presented here. Moreover, although hospital data were objective, barriers to care were self-reported. It is possible that those with lower SES may report differently. In addition, it is unknown what percentage of World Trade Center Health Registry enrollees are enrolled or engage in asthma management in the WTCHP. Also, hospital data cover only New York State, despite the fact that a considerable number of enrollees did not reside in New York State on 9/11 or have since moved. For these reasons, the results may not be generalizable to the entire 9/11-exposed cohort or the general population.

## Conclusions

Barriers to care appear to explain a relatively modest portion of current asthma-related health disparities in this cohort, but it is clear that further research is needed. Health equity requires that public health officials identify vulnerable postdisaster subpopulations and provide additional resources to those who most need them.

## Notes

Supplement. **eTable 1.** Study Characteristics by Any Barrier to Care **eTable 2.** Direct and Indirect Effect Sizes of Race on Number of Both Asthma ED Visits and Inpatient Stays **eTable 3.** Direct and Indirect Effect Sizes of Income on Number of Both Asthma ED Visits and Inpatient Stays **eTable 4.** Direct and Indirect Effect Sizes of Education on Number of Both Asthma ED Visits and Inpatient Stays **eTable 5.** Direct and Indirect Effect Sizes of Race on Number of Asthma Inpatient Stays **eTable 6.** Direct and Indirect Effect Sizes of Income on Number of Asthma Inpatient Stays **eTable 7.** Direct and Indirect Effect Sizes of Education on Number of Asthma Inpatient Stays **eTable 8.** Direct and Indirect Effect Sizes of Race on Number of Asthma ED Visits Among Self-reported Asthmatics **eTable 9.** Direct and Indirect Effect Sizes of Income on Number of Asthma ED Visits Among Self-reported Asthmatics **eTable 10.** Direct and Indirect Effect Sizes of Education on Number of Asthma ED Visits Among Self-reported Asthmatics
