# Supplementary material for: Association Between Socioeconomic Status and Asthma-Related Emergency Department Visits Among World Trade Center Rescue and Recovery Workers and Survivors
Source: JAMA Netw Open. 2020 Mar 23;3(3):e201600. doi: 10.1001/jamanetworkopen.2020.1600 (PMC7090833; doi:10.1001/jamanetworkopen.2020.1600)
Supplement: Supplement. — eTable 1. Study Characteristics by Any Barrier to Care eTable 2. Direct and Indirect Effect Sizes of Race on Number of Both Asthma ED Visits and Inpatient Stays eTable 3. Direct and Indirect Effect Sizes of Income on Number of Both Asthma ED Visits and Inpatient Stays eTable 4. Direct and Indirect Effect Sizes of Education on Number of Both Asthma ED Visits and Inpatient Stays eTable 5. Direct and Indirect Effect Sizes of Race on Number of Asthma Inpatient Stays eTable 6. Direct and Indirect Effect Sizes of Income on Number of Asthma Inpatient Stays eTable 7. Direct and Indirect Effect Sizes of Education on Number of Asthma Inpatient Stays eTable 8. Direct and Indirect Effect Sizes of Race on Number of Asthma ED Visits Among Self-reported Asthmatics eTable 9. Direct and Indirect Effect Sizes of Income on Number of Asthma ED Visits Among Self-reported Asthmatics eTable 10. Direct and Indirect Effect Sizes of Education on Number of Asthma ED Visits Among Self-reported Asthmatics [file jamanetwopen-3-e201600-s001.pdf]

# Supplementary Online Content

Brite J, Alper H, Friedman S, Takemoto E, Cone J. Association between socioeconomic status and asthma-related emergency department visits among World Trade Center rescue and recovery workers and survivors. *JAMA Netw Open*. 2020;3(3):e201600. doi:10.1001/jamanetworkopen.2020.1600

**eTable 1.** Study Characteristics by Any Barrier to Care

**eTable 2.** Direct and Indirect Effect Sizes of Race on Number of Both Asthma ED Visits and Inpatient Stays

**eTable 3.** Direct and Indirect Effect Sizes of Income on Number of Both Asthma ED Visits and Inpatient Stays

**eTable 4.** Direct and Indirect Effect Sizes of Education on Number of Both Asthma ED Visits and Inpatient Stays

**eTable 5.** Direct and Indirect Effect Sizes of Race on Number of Asthma Inpatient Stays

**eTable 6.** Direct and Indirect Effect Sizes of Income on Number of Asthma Inpatient Stays

**eTable 7.** Direct and Indirect Effect Sizes of Education on Number of Asthma Inpatient Stays

**eTable 8.** Direct and Indirect Effect Sizes of Race on Number of Asthma ED Visits Among Self-reported Asthmatics

**eTable 9.** Direct and Indirect Effect Sizes of Income on Number of Asthma ED Visits Among Self-reported Asthmatics

**eTable 10.** Direct and Indirect Effect Sizes of Education on Number of Asthma ED Visits Among Self-reported Asthmatics

This supplementary material has been provided by the authors to give readers additional information about their work.

© 2020 Brite J et al. *JAMA Network Open.*

## eTable 1: Study characteristics by any barrier to care

| | *Any Barrier to Care* | | | |
|---|---|---|---|---|
| | *Overall (N=30452)* | *Yes (N=5967)* | *No (N=24485)* | *p-value* |
| ***Exposures*** | | | | |
| **Race** | | | | <0.001 |
| **White** | 20180 (66%) | 3394 (57%) | 16786 (69%) | |
| **Black or African American** | 3834 (13%) | 835 (14%) | 2999 (12%) | |
| **Hispanic or Latino (any race)** | 3961 (13%) | 1117 (19%) | 2844 (12%) | |
| **Asian (includes Native Hawaiian/Pacific Islander** | 1697 (6%) | 416 (7%) | 1281 (5%) | |
| **Multiracial/Other** | 780 (3%) | 205 (3%) | 575 (2%) | |
| **Education** | | | | <0.001 |
| **Less than high school** | 1276 (4%) | 435 (7%) | 841 (3%) | |
| **High school only** | 5914 (19%) | 1377 (23%) | 4537 (19%) | |
| **Some college** | 7679 (25%) | 1659 (28%) | 6020 (25%) | |
| **At least a Bachelor's** | 15583 (51%) | 2496 (42%) | 13087 (53%) | |
| **Income** | | | | <0.001 |
| **Less than $35,000** | 4537 (15%) | 1538 (26%) | 2999 (12%) | |
| **$35,000 to less than $75,000** | 10691 (35%) | 2194 (37%) | 8497 (35%) | |
| **$75,000 to less than $200,000** | 13139 (43%) | 2058 (34%) | 11081 (45%) | |
| **$200,000 or more** | 2085 (7%) | 177 (3%) | 1908 (8%) | |
| ***Covariates*** | | | | |
| **Gender** | | | | <0.001 |
| **Male** | 18585 (61%) | 3495 (59%) | 15090 (62%) | |
| **Female** | 11867 (39%) | 2472 (41%) | 9395 (38%) | |
| **Age (yr)** | 42.0 (35.0, 50.0) | 41.0 (34.0, 48.0) | 42.0 (35.0, 50.0) | <0.001 |
| **Self-reported asthma** | | | | <0.001 |
| **Yes** | 5897 (19%) | 1471 (25%) | 4426 (18%) | |
| **No** | 24330 (80%) | 4407 (75%) | 19923 (82%) | |
| **Don't know** | 19 (0%) | 4 (0%) | 15 (0%) | |
| **Refused** | 1 (0%) | 1 (0%) | 0 (0%) | |

Note: Values expressed as N(%), mean ± standard deviation or median ($25^{th}$,$75^{th}$ percentiles)
P-value comparisons across treatment groups for categorical variables are based on chi-square test of homogeneity; p-values for continuous variables are based on ANOVA or Kruskal-Wallis test for median

© 2020 Brite J et al. *JAMA Network Open.*

eTable 2. Direct and indirect effect sizes of race on number of both asthma ED visits and inpatient stays

| **RACE** | | | |
|---|---|---|---|
| | ***Rates of* ED visits and inpatient stays *per 100 enrollees*** | | |
| White | 1.7(1.6, 2.0) | | |
| African American | 17.1 (15.6, 18.7) | | |
| Hispanic | 8.4 (7.5, 9.5) | | |
| Asian | 1.0 (0.7, 1.6) | | |
| Other | 4.9 (3.6, 6.6) | | |
| | **Change in number of ED visits and inpatient stays per 100** | ***p-value*** | **% Mediated (95% CI) [a]** |
| *African Americans v. Whites* | | | |
| Total effect | 15.6 (14.2, 17.0) | <0.001 * | |
| Indirect effects | | | |
| Lack of money | 0.7 (0.4, 0.9) | <0.001 * | 4.0 (2.8, 5.3) |
| Lack insurance | 0.7 (0.4, 0.9) | <0.001 * | 3.9 (2.8, 5.5) |
| Lack transportation | 0.8 (0.5, 1.2) | <0.001 * | 4.9 (3.2, 7.1) |
| Lack of childcare | 0.0 (0.0, 0.0) | 0.39 | 0.0 (0.0, 0.2) |
| Didn't know where to go for care | 0.1 (0.0, 0.2) | 0.05 | 0.7 (0.0, 1.4) |
| Unable to find a provider | 0.0 (-0.1, 0.0) | 0.36 | -0.1 (-0.3, 0.2) |
| Number of barriers | 0.5 (0.4, 0.6) | <0.001 * | 3.2 (2.6, 3.9) |
| *Hispanic v. Whites* | | | |
| Total effect | 6.8 (5.9, 7.8) | <0.001 * | |
| Indirect effects | | | |
| Lack of money | 0.5 (0.4, 0.6) | <0.001 * | 6.8 (5.2, 8.6) |
| Lack insurance | 0.5 (0.4, 0.6) | <0.001 * | 6.8 (5.2, 8.7) |
| Lack transportation | 0.4 (0.3, 0.6) | <0.001 * | 5.6 (3.7, 8.1) |
| Lack of childcare | 0.0 (0.0, 0.0) | 0.42 | 0.0 (-0.2, 0.1) |
| Didn't know where to go for care | 0.2 (0.1, 0.3) | <0.001 * | 2.7 (1.7, 4.1) |
| Unable to find a provider | 0.0 (0.0, 0.0) | 0.36 | -0.2 (-0.6, 0.3) |
| Number of barriers | 0.5 (0.4, 0.6) | <0.001 * | 7.8 (6.7, 9.1) |
| *Asian v. Whites [b]* | | | |
| Total effect | -0.7 (-1.2, -0.2) | 0.03 * | |
| Indirect effects | | | |
| Lack of money | 0.1 (0.0, 0.1) | 0.03 * | |
| Lack insurance | 0.1 (0.0, 0.1) | 0.03 * | |
| Lack transportation | 0.1 (0.0, 0.1) | 0.01 * | |
| Lack of childcare | 0.0 (0.0, 0.0) | 0.48 | |
| Didn't know where to go for care | 0.0 (0.0, 0.1) | 0.02 * | |
| Unable to find a provider | 0.0 (0.0, 0.0) | 0.42 | |
| Number of barriers | 0.1 (0.1, 0.1) | <0.001 * | |
| *Other v. Whites* | | | |
| Total effect | 3.1 (1.9, 4.7) | <0.001 * | |
| Indirect effects | | | |
| Lack of money | 0.5 (0.3, 0.7) | <0.001 * | 13.9 (9.2, 21.3) |
| Lack insurance | 0.5 (0.3, 0.7) | <0.001 * | 13.8 (9.0, 21.6) |
| Lack transportation | 0.5 (0.3, 0.9) | <0.001 * | 14.5 (8.5, 24.8) |
| Lack of childcare | 0.0 (0.0, 0.0) | 0.40 | -0.1 (-0.8, 0.2) |
| Didn't know where to go for care | 0.1 (0.0, 0.2) | 0.18 | 1.5 (-0.5, 4.6) |

© 2020 Brite J et al. *JAMA Network Open.*

| | | | |
|---|---|---|---|
| Unable to find a provider | 0.0 (0.0, 0.0) | 0.4 | -0.1 (-0.7, 0.3) |
| Number of barriers | 0.4 (0.3, 0.5) | <0.001 * | 11.3 (8.0, 17.0) |
| | | | |

All estimates adjusted by age and gender.
* Significant at the <0.05 level.

a. Percent mediated = indirect effect/(direct effect + indirect effect) × 100
b. Percent mediated not calculated for Asians because lower rates of hospitalization than Whites.

© 2020 Brite J et al. *JAMA Network Open.*

eTable 3. Direct and indirect effect sizes of income on number of both asthma ED visits and inpatient stays

| **Income** | | | |
|---|---|---|---|
| | ***Rates of*** **ED visits and inpatient stays** ***per 100 enrollees*** | | |
| Less than $35,000 | 14.4 (13.2, 15.7) | | |
| $35,000 to less than $75,000 | 3.8 (3.5, 4.3) | | |
| $75,000 to less than $200,000 | 1.8 (1.5, 2.0) | | |
| $200,000 or more | 0.5 (0.3, 0.8) | | |
| | **Change in number of ED visits and inpatient stays per 100** | ***p-value*** | **% Mediated (95% CI)** [a] |
| *Less than $35,000 v. $200,000 or more* | | | |
| Total effect | 15.6 (14.5, 16.9) | <0.001 * | |
| Indirect effects | | | |
| Lack of money | 1.5 (1.2, 1.8) | <0.001 * | 8.9 (7.3, 10.8) |
| Lack insurance | 1.5 (1.2, 1.8) | <0.001 * | 9.0 (7.3, 11.0) |
| Lack transportation | 1.2 (0.9, 1.6) | <0.001 * | 7.4 (5.7, 9.3) |
| Lack of childcare | 0.0 (0.0, 0.0) | 0.22 | -0.1 (-0.3, 0.0) |
| Didn't know where to go for care | 0.5 (0.3, 0.7) | <0.001 * | 2.9 (2.0, 4.1) |
| Unable to find a provider | -0.1 (-0.2, 0.0) | 0.06 | -0.6 (-1.2, 0.1) |
| Number of barriers | 1.6 (1.3, 1.8) | <0.001 * | 10.0 (8.5, 11.7) |
| *$35,000 to less than $75,000 v. $200,000 or more* | | | |
| Total effect | 3.8 (3.3, 4.3) | <0.001 * | |
| Indirect effects | | | |
| Lack of money | 0.2 (0.1, 0.2) | <0.001 * | 4.4 (3.4, 5.9) |
| Lack insurance | 0.2 (0.1, 0.2) | <0.001 * | 4.0 (2.9, 5.5) |
| Lack transportation | 0.1 (0.0, 0.1) | 0.01 * | 1.6 (0.7, 2.5) |
| Lack of childcare | 0.0 (0.0, 0.0) | 0.29 | -0.1 (-0.2, 0.1) |
| Didn't know where to go for care | 0.1 (0.0, 0.1) | <0.001 * | 1.5 (0.9, 2.3) |
| Unable to find a provider | 0.0 (0.0, 0.0) | 0.03 * | -0.3 (-0.6, 0.0) |
| Number of barriers | 0.2 (0.2, 0.2) | <0.001 * | 4.7 (3.9, 5.9) |
| *$75,000 to less than $200,000 v. $200,000 or more* | | | |
| Total effect | 1.5 (1.1, 1.9) | <0.001 * | |
| Indirect effects | | | |
| Lack of money | 0.0 (0.0, 0.0) | <0.001 * | 1.8 (0.9, 3.2) |
| Lack insurance | 0.0 (0.0, 0.0) | 0.01 * | 1.2 (0.4, 2.5) |
| Lack transportation | 0.0 (0.0, 0.0) | 0.47 | 0.3 (-0.9, 1.1) |
| Lack of childcare | 0.0 (0.0, 0.0) | 0.17 | -0.2 (-0.4, 0.1) |
| Didn't know where to go for care | 0.0 (0.0, 0.0) | 0.002 * | 1.1 (0.5, 2.2) |
| Unable to find a provider | 0.0 (0.0, 0.0) | 0.04 * | -0.2 (-0.6, 0.0) |
| Number of barriers | 0.1 (0.0, 0.1) | <0.001 * | 3.8 (2.1, 5.3) |

All estimates adjusted by age and gender.
* Significant at the <0.05 level.
a. Percent mediated = indirect effect/(direct effect + indirect effect) × 100

eTable 4. Direct and indirect effect sizes of education on number of both asthma ED visits and inpatient stays

| Education | | | |
|---|---|---|---|
| | Rate of ED visits and inpatient stays per 100 enrollees | | |
| At least a Bachelor's | 1.7 (1.5, 1.9) | | |
| Some college | 6.6 (5.6, 7.4) | | |
| High school | 6.6 (6.0, 7.4) | | |
| Less than high school | 11.5 (9.8, 13.6) | | |
| | **Change in number of ED visits and inpatient stays per 100** | ***p-value*** | **% Mediated (95% CI) [a]** |
| Some college v. at least a Bachelor's | | | |
| Total effect | 5.5 (4.9, 6.2) | <0.001 * | |
| Indirect effects | | | |
| Lack of money | 0.2 (0.1, 0.3) | <0.001 * | 3.9 (2.5, 5.6) |
| Lack insurance | 0.2 (0.1, 0.3) | 0.002 * | 3.1 (1.4, 5.0) |
| Lack transportation | 0.4 (0.2, 0.5) | <0.001 * | 6.7 (4.2, 9.8) |
| Lack of childcare | 0.0 (0.0, 0.0) | 0.05 | -0.2 (-0.4, 0.0) |
| Didn't know where to go for care | 0.1 (0.1, 0.2) | <0.001 * | 1.7 (1.1, 2.8) |
| Unable to find a provider | 0.0 (0.0, 0.0) | 0.44 | -0.1 (-0.3, 0.1) |
| Number of barriers | 0.2 (0.2, 0.3) | <0.001 * | 4.3 (3.3, 5.3) |
| High school v. at least a Bachelor's | | | |
| Total effect | 5.5 (4.8, 6.2) | <0.001 * | |
| Indirect effects | | | |
| Lack of money | 0.3 (0.2, 0.4) | <0.001 * | 6.1 (4.1, 8.3) |
| Lack insurance | 0.3 (0.2, 0.4) | <0.001 * | 5.8 (3.7, 8.2) |
| Lack transportation | 0.2 (0.1, 0.4) | <0.001 * | 4.1 (2.0, 7.3) |
| Lack of childcare | 0.0 (0.0, 0.0) | 0.05 | -0.3 (-0.6, 0.0) |
| Didn't know where to go for care | 0.1 (0.1, 0.2) | <0.001 * | 1.8 (1.0, 2.8) |
| Unable to find a provider | 0.0 (0.0, 0.0) | 0.47 | -0.1 (-0.3, 0.2) |
| Number of barriers | 0.3 (0.3, 0.4) | <0.001 * | 4.7 (3.8, 5.8) |
| Less than high school v. at least a Bachelor's | | | |
| Total effect | 10.7 (8.9, 12.8) | <0.001 * | |
| Indirect effects | | | |
| Lack of money | 1.1 (0.7, 2.0) | <0.001 * | 11.0 (7.5, 15.1) |
| Lack insurance | 1.1 (0.8, 2.3) | <0.001 * | 11.4 (8.0, 16.0) |
| Lack transportation | 0.6 (0.2, 1.2) | <0.001 * | 5.7 (2.2, 10.7) |
| Lack of childcare | 0.0 (-0.1, 0.0) | 0.13 | -0.2 (-0.6, 0.0) |
| Didn't know where to go for care | 0.5 (0.3, 0.8) | <0.001 * | 4.4 (2.8, 7.4) |
| Unable to find a provider | 0.0 (-0.1, 0.1) | 0.5 | -0.3 (-1.0, 0.6) |
| Number of barriers | 1.0 (0.7, 1.2) | <0.001 * | 9.1 (7.3, 10.9) |
| | | | |
| All estimates adjusted by age and gender. | | | |

* Significant at the <0.05 level.

a. Percent mediated = indirect effect/(direct effect + indirect effect) × 100

© 2020 Brite J et al. *JAMA Network Open.*

eTable 5. Direct and indirect effect sizes of race on number of asthma inpatient stays

| **RACE** | | | |
|---|---|---|---|
| ***Rates of* inpatient stays *per 100 enrollees*** | | | |
| White | 0.6 (0.6, 0.8) | | |
| African American | 4.4 (3.7, 5.3) | | |
| Hispanic | 2.4 (1.9, 3.1) | | |
| Asian | 0.3 (0.1, 0.8) | | |
| Other | 0.8 (0.4, 1.9) | | |
| | **Change in number of inpatient stays per 100** | ***p-value*** | **% Mediated (95% CI)** [a] |
| *African Americans v. Whites* | | | |
| Total effect | 3.2 (2.6, 3.8) | <0.001 * | |
| Indirect effects | | | |
| Lack of money | 0.1 (0.0, 0.2) | <0.001 * | 4.1 (2.5, 6.4) |
| Lack insurance | 0.1 (0.1, 0.2) | <0.001 * | 4.2 (2.6, 6.5) |
| Lack transportation | 0.4 (0.2, 0.6) | <0.001 * | 10.5 (6.7, 15.2) |
| Lack of childcare | 0.0 (0.0, 0.0) | 0.52 | 0.0 (-0.2, 0.2) |
| Didn't know where to go for care | 0.0 (0.0, 0.0) | 0.16 | 0.2 (-0.5, 0.1) |
| Unable to find a provider | 0.0 (0.0, 0.0) | 0.18 | -0.4 (-0.8, 0.2) |
| Number of barriers | 0.1 (0.1, 0.1) | <0.001 * | 2.9 (1.9, 4.2) |
| *Hispanic v. Whites* | | | |
| Total effect | 1.5 (1.1, 2.1) | <0.001 * | |
| Indirect effects | | | |
| Lack of money | 0.1 (0.1, 0.2) | <0.001 * | 7.6 (4.8, 11.5) |
| Lack insurance | 0.1 (0.1, 0.2) | <0.001 * | 7.8 (5.1, 11.8) |
| Lack transportation | 0.2 (0.1, 0.3) | <0.001 * | 12.6 (7.6, 19.1) |
| Lack of childcare | 0.0 (0.0, 0.0) | 0.50 | 0.1 (-0.3, 0.2) |
| Didn't know where to go for care | 0.0 (0.0, 0.0) | 0.11 | -0.9 (-1.7, 0.3) |
| Unable to find a provider | 0.0 (0.0, 0.0) | 0.17 | -0.7 (-1.6, 0.3) |
| Number of barriers | 0.1 (0.1, 0.1) | <0.001 * | 6.8 (4.7, 9.7) |
| *Asian v. Whites* [b] | | | |
| Total effect | -0.3 (-0.5, 0.1) | 0.12 | |
| Indirect effects | | | |
| Lack of money | 0.0 (0.0, 0.1) | <0.001 * | |
| Lack insurance | 0.0 (0.0, 0.0) | <0.001 * | |
| Lack transportation | 0.0 (0.0, 0.0) | 0.01 * | |
| Lack of childcare | 0.0 (0.0, 0.1) | 0.58 | |
| Didn't know where to go for care | 0.0 (0.0, 0.0) | 0.1 | |
| Unable to find a provider | 0.0 (0.0, 0.0) | 0.17 | |
| Number of barriers | 0.0 (0.0, 0.0) | <0.001 * | |
| *Other v. Whites* | | | |
| Total effect | 0.2 (-0.3, 1.2) | 0.64 | |
| Indirect effects | | | |
| Lack of money | 0.1 (0.0, 0.2) | <0.001 * | 24.3 (-354.6, 399.7) |
| Lack insurance | 0.1 (0.0, 0.2) | <0.001 * | 23.2 (-562.6, 483.2) |
| Lack transportation | 0.2 (0.1, 0.4) | <0.001 * | 47.7 (-699.1, 1128.4) |
| Lack of childcare | 0.0 (0.0, 0.0) | 0.55 | 0.0 (-8.1, 9.1) |
| Didn't know where to go for care | 0.0 (0.0, 0.0) | 0.12 | 0.4 (-11.8, 13.7) |
| Unable to find a provider | 0.0 (0.0, 0.0) | 0.16 | -0.7 (-18.4, 23.0) |
| Number of barriers | 0.1 (0.0, 0.1) | <0.001 * | 14.5 (-177.2, 290.8) |

© 2020 Brite J et al. *JAMA Network Open.*

| | | | |
|---|---|---|---|
| All estimates adjusted by age and gender.<br>* Significant at the <0.05 level. | | | |
| a. Percent mediated = indirect effect/(direct effect + indirect effect) × 100<br>b. Percent mediated not calculated for Asians because lower rates of hospitalization than Whites. | | | |

© 2020 Brite J et al. *JAMA Network Open.*

eTable 6. Direct and indirect effect sizes of income on number of asthma inpatient stays

| **Income** | | | |
|---|---|---|---|
| ***Rates of* inpatient stays *per 100 enrollees*** | | | |
| Less than $35,000 | 4.1 (3.5, 4.8) | | |
| $35,000 to less than $75,000 | 0.8 (0.6, 1.0) | | |
| $75,000 to less than $200,000 | 0.7 (0.6, 0.9) | | |
| $200,000 or more | 0.1 (0.0, 0.4) | | |
| | **Change in number of inpatient stays per 100** | ***p-value*** | **% Mediated (95% CI) [a]** |
| *Less than $35,000 v. $200,000 or more* | | | |
| Total effect | 3.9 (3.3, 4.6) | <0.001 * | |
| Indirect effects | | | |
| Lack of money | 0.3 (0.2, 0.5) | <0.001 * | 8.5 (5.3, 12.8) |
| Lack insurance | 0.4 (0.2, 0.5) | <0.001 * | 8.8 (5.6, 13.1) |
| Lack transportation | 0.5 (0.4, 0.8) | <0.001 * | 13.3 (9.7, 18.2) |
| Lack of childcare | 0.0 (0.0, 0.0) | 0.31 | -0.1 (-0.4, 0.2) |
| Didn't know where to go for care | -0.1 (-0.1, 0.0) | 0.02 * | -1.5 (-2.3, -0.3) |
| Unable to find a provider | 0.0 (-0.1, 0.0) | 0.04 * | -1.2 (-2.1, 0.1) |
| Number of barriers | 0.3 (0.2, 0.4) | <0.001 * | 7.5 (5.1, 10.5) |
| *$35,000 to less than $75,000 v. $200,000 or more* | | | |
| Total effect | 0.6 (0.3, 0.9) | 0.01 * | |
| Indirect effects | | | |
| Lack of money | 0.0 (0.0, 0.1) | 0.004 * | 4.8 (2.6, 12.1) |
| Lack insurance | 0.0 (0.0, 0.0) | <0.001 * | 4.5 (2.4, 10.6) |
| Lack transportation | 0.0 (0.0, 0.0) | 0.02 * | 3.5 (1.1, 9.7) |
| Lack of childcare | 0.0 (0.0, 0.0) | 0.45 | -0.1 (-0.4, 0.3) |
| Didn't know where to go for care | 0.0 (0.0, 0.0) | 0.02 * | -0.9 (-2.5, -0.2) |
| Unable to find a provider | 0.0 (0.0, 0.0) | 0.05 | -0.7 (-1.8, 0.0) |
| Number of barriers | 0.0 (0.0, 0.0) | <0.001 * | 4.4 (2.5, 10.7) |
| *$75,000 to less than $200,000 v. $200,000 or more* | | | |
| Total effect | 0.6 (0.3, 0.8) | 0.006 * | |
| Indirect effects | | | |
| Lack of money | 0.0 (0.0, 0.0) | <0.001 * | 1.4 (0.6, 3.8) |
| Lack insurance | 0.0 (0.0, 0.0) | 0.004 * | 1.1 (0.4, 3.5) |
| Lack transportation | 0.0 (0.0, 0.0) | 0.43 | 0.5 (-1.9, 2.1) |
| Lack of childcare | 0.0 (0.0, 0.0) | 0.35 | -0.2 (-0.7, 0.7) |
| Didn't know where to go for care | 0.0 (0.0, 0.0) | 0.02 * | -0.4 (-1.2, -0.1) |
| Unable to find a provider | 0.0 (0.0, 0.0) | 0.07 | -0.4 (-1.2, 0.1) |
| Number of barriers | 0.0 (0.0, 0.0) | <0.001 * | 2.1 (1.2, 5.5) |
| | | | |
| All estimates adjusted by age and gender.<br>* Significant at the <0.05 level. | | | |
| a. Percent mediated = indirect effect/(direct effect + indirect effect) × 100 | | | |

© 2020 Brite J et al. *JAMA Network Open.*

eTable 7. Direct and indirect effect sizes of education on number of asthma inpatient stays

| **Education** | | | |
|---|---|---|---|
| | **Rate of inpatient stays per 100 enrollees** | | |
| At least a Bachelor's | 0.4 (0.3, 0.6) | | |
| Some college | 1.2 (0.9, 1.5) | | |
| High school | 3.0 (2.4, 3.6) | | |
| Less than high school | 3.8 (2.9, 5.1) | | |
| | **Change in number of inpatient stays per 100** | ***p-value*** | **% Mediated (95% CI)** [a] |
| Some college v. at least a Bachelor's | | | |
| Total effect | 0.7(0.5, 1.0) | <0.001 * | |
| Indirect effects | | | |
| Lack of money | 0.2 (0.1, 0.3) | <0.001 * | 4.5 (2.8, 6.6) |
| Lack insurance | 0.2 (0.1, 0.3) | 0.002 * | 3.1 (1.2, 5.3) |
| Lack transportation | 0.3 (0.1, 0.4) | <0.001 * | 4.8 (4.2, 9.8) |
| Lack of childcare | 0.0 (0.0, 0.0) | 0.24 | -0.2 ( -0.6, 0.5) |
| Didn't know where to go for care | 0.0 (-0.1, 0.0) | 0.07 | -0.7 (-1.5, 0.1) |
| Unable to find a provider | 0.0 (0.0, 0.0) | 0.13 | -0.3 (-0.9, 0.2) |
| Number of barriers | 0.00 (0.0, 0.0) | <0.001 * | 4.5 (2.9, 7.1) |
| High school v. at least a Bachelor's | | | |
| Total effect | 2.4 (2.0, 2.8) | <0.001 * | |
| Indirect effects | | | |
| Lack of money | 0.1 (0.1, 0.2) | <0.001 * | 4.1 (2.5, 6.6) |
| Lack insurance | 0.1 (0.1, 0.2) | <0.001 * | 4.0 (2.3, 6.6) |
| Lack transportation | 0.2 (0.1, 0.3) | <0.001 * | 6.6 (3.2, 11.5) |
| Lack of childcare | 0.0 (0.0, 0.0) | 0.18 | -0.3 (-0.6, 0.4) |
| Didn't know where to go for care | 0.0 (0.0, 0.0) | 0.06 | -0.5 (-0.9, 0.0) |
| Unable to find a provider | 0.0 (0.0, 0.0) | 0.13 | -0.2 (-0.5, 0.1) |
| Number of barriers | 0.1 (0.0, 0.1) | <0.001 * | 3.1 (2.1, 4.1) |
| Less than high school v. at least a Bachelor's | | | |
| Total effect | 3.2 (2.2, 4.4) | <0.001 * | |
| Indirect effects | | | |
| Lack of money | 0.3 (0.2, 0.6) | <0.001 * | 8.9 (5.5, 13.8) |
| Lack insurance | 0.3 (0.2, 0.6) | <0.001 * | 9.6 (5.8, 14.6) |
| Lack transportation | 0.4 (0.1, 0.8) | 0.002 * | 9.9 (4.0, 18.6) |
| Lack of childcare | 0.0 (0.0, 0.0) | 0.27 | -0.1 (-0.6, 0.1) |
| Didn't know where to go for care | 0.6 (0.4, 1.0) | 0.06 | 7.1 (4.5, 10.9) |
| Unable to find a provider | 0.0 (-0.1, 0.1) | 0.16 | 0.0 (-0.8, 1.0) |
| Number of barriers | 0.2 (0.1, 0.3) | <0.001 * | 6.8 (4.9, 9.1) |
| | | | |
| All estimates adjusted by age and gender. | | | |
| * Significant at the <0.05 level. | | | |
| a. Percent mediated = indirect effect/(direct effect + indirect effect) × 100 | | | |

© 2020 Brite J et al. *JAMA Network Open.*

eTable 8. Direct and indirect effect sizes of race on number of asthma ED visits among self-reported asthmatics

| **RACE** | | | |
|---|---|---|---|
| ***Rates of* ED visits *per 100 enrollees*** | | | |
| White | 4.2 (3.6, 4.9) | | |
| African American | 60.1 (54.4, 66.4) | | |
| Hispanic | 18.8 (16.3, 21.7) | | |
| Asian | 4.0 (2.3, 6.9) | | |
| Other | 16.3 (11.5, 23.1) | | |
| | **Change in number of ED visits per 100** | ***p-value*** | **% Mediated (95% CI)** [a] |
| *African Americans v. Whites* | | | |
| Total effect | 62.3 (56.1, 68.7) | <0.001 * | |
| Indirect effects | | | |
| Lack of money | 2.3 (1.1, 3.9) | <0.001 * | 3.4 (1.7, 5.6) |
| Lack insurance | 2.6 (1.1, 4.6) | <0.001 * | 3.9 (1.8, 6.7) |
| Lack transportation | 2.4 (1.1, 4.3) | <0.001 * | 3.5 (1.7, 6.4) |
| Lack of childcare | 0.0 (-0.2, 0.2) | 0.76 | 0.0 (-0.3, 0.3) |
| Didn't know where to go for care | 0.9 (-0.4, 2.7) | 0.21 | 1.3 (-0.7, 3.9) |
| Unable to find a provider | -0.1 (-0.3, 0.1) | 0.37 | -0.1 (-0.5, 0.1) |
| Number of barriers | 2.0 (1.1, 3.0) | <0.001 * | 3.1 (1.8, 4.8) |
| *Hispanic v. Whites* | | | |
| Total effect | 16.2 (13.3, 19.3) | <0.001 * | |
| Indirect effects | | | |
| Lack of money | 1.2 (0.7, 1.4) | <0.001 * | 6.7 (4.1, 9.9) |
| Lack insurance | 0.9 (0.4, 1.6) | <0.001 * | 5.2 (2.5, 8.6) |
| Lack transportation | 0.6 (0.2, 1.1) | <0.001 * | 3.2 (1.1, 6.3) |
| Lack of childcare | -0.1 (-0.2, 0.0) | 0.20 | -0.3 (-1.0, 0.2) |
| Didn't know where to go for care | 0.7 (0.2, 1.3) | 0.002 * | 4.1 (1.3, 7.3) |
| Unable to find a provider | 0.0 (-0.1, 0.0) | 0.33 | -0.1 (-0.7, 0.2) |
| Number of barriers | 0.9 (0.6, 1.3) | <0.001 * | 5.8 (4.0, 8.0) |
| *Asian v. Whites* [b] | | | |
| Total effect | -0.2 (-2.5, 2.8) | 0.80 | |
| Indirect effects | | | |
| Lack of money | 0.4 (0.2, 0.8) | <0.001 * | |
| Lack insurance | 0.4 (0.1, 0.9) | <0.001 * | |
| Lack transportation | 0.2 (0.0, 0.6) | 0.018 * | |
| Lack of childcare | 0.0 (-0.1, 0.0) | 0.74 | |
| Didn't know where to go for care | 0.3 (0.0, 0.8) | 0.02 * | |
| Unable to find a provider | 0.0 (0.0, 0.0) | 0.31 | |
| Number of barriers | 0.3 (0.2, 0.6) | <0.001 * | |
| *Other v. Whites* | | | |
| Total effect | 13.9 (8.3, 21.0) | <0.001 * | |
| Indirect effects | | | |
| Lack of money | 1.7 (0.8, 2.9) | <0.001 * | 11.4 (6.3, 18.9) |
| Lack insurance | 1.7 (0.7, 3.0) | <0.001 * | 11.4 (5.4, 19.0) |
| Lack transportation | 0.8 (0.1, 2.2) | 0.01 * | 4.5 (0.8, 13.1) |
| Lack of childcare | -0.1 (-0.3, 0.1) | 0.52 | -0.2 (-2.2, 0.3) |
| Didn't know where to go for care | -0.2 (-0.8, 0.7) | 0.48 | -1.9 (-5.7, 4.4) |

© 2020 Brite J et al. *JAMA Network Open.*

| | | | |
|---|---|---|---|
| Unable to find a provider | 0.0 (-0.1, 0.1) | 0.99 | 0.0 (-0.9, 0.5) |
| Number of barriers | 0.9 (0.4, 1.6) | 0.002 * | 6.5 (2.9, 11.6) |
| | | | |
| All estimates adjusted by age and gender.<br>* Significant at the <0.05 level. | | | |
| | | | |
| a. Percent mediated = indirect effect/(direct effect + indirect effect) × 100<br>b. Percent mediated not calculated for Asians because lower rates of hospitalization than Whites. | | | |

© 2020 Brite J et al. *JAMA Network Open.*

eTable 9. Direct and indirect effect sizes of income on number of asthma ED visits among self-reported asthmatics

| **Income** | | | |
|---|---|---|---|
| | ***Rates of* ED visits *per 100 enrollees*** | | |
| Less than $35,000 | 44.4 (40.1, 49.0) | | |
| $35,000 to less than $75,000 | 12.8 (11.4, 14.5) | | |
| $75,000 to less than $200,000 | 4.3 (3.6, 5.2) | | |
| $200,000 or more | 1.5 (0.7, 3.3) | | |
| | **Change in number of ED visits per 100** | ***p-value*** | **% Mediated (95% CI) [a]** |
| *Less than $35,000 v. $200,000 or more* | | | |
| Total effect | 49.1 (43.8, 54.2) | <0.001 * | |
| Indirect effects | | | |
| Lack of money | 3.7 (2.5, 5.2) | <0.001 * | 7.3 (5.0, 10.0) |
| Lack insurance | 4.3 (2.9, 5.9) | <0.001 * | 8.4 (5.8, 11.5) |
| Lack transportation | -22.9 (-63.1, 4.3) | 0.999 | -3.7 (-144.3, 8.1) |
| Lack of childcare | -0.1 (-0.4, 0.3) | 0.62 | -0.1 (-0.7, 0.6) |
| Didn't know where to go for care | 2.6 (1.5, 4.0) | <0.001 * | 5.0 (2.9, 7.6) |
| Unable to find a provider | -0.6 (-0.9, 0.0) | 0.02 * | -1.1 (-1.9, -0.2) |
| Number of barriers | 4.6 (3.6, 5.8) | <0.001 * | 9.4 (7.4, 11.7) |
| *$35,000 to less than $75,000 v. $200,000 or more* | | | |
| Total effect | 13.3 (10.8, 15.5) | <0.001 * | |
| Indirect effects | | | |
| Lack of money | 0.6 (0.3, 0.9) | <0.001 * | 4.3 (2.5, 6.6) |
| Lack insurance | 0.6 (0.3, 0.8) | <0.001 * | 4.2 (2.1, 6.5) |
| Lack transportation | -8.0 (-23.7, 0.3) | 0.92 | 0.9 (-419.6, 2.6) |
| Lack of childcare | 0.0 (0.0, 0.1) | 0.52 | 0.1 (-0.2, 1.1) |
| Didn't know where to go for care | 0.3 (0.0, 0.5) | 0.03 * | 2.1 (0.2, 3.9) |
| Unable to find a provider | -0.1 (-0.2, 0.0) | 0.03 * | -0.7 (-1.4, -0.1) |
| Number of barriers | 0.7 (0.6, 1.0) | <0.001 * | 5.3 (3.9, 7.8) |
| *$75,000 to less than $200,000 v. $200,000 or more* | | | |
| Total effect | 3.2 (1.1, 4.8) | 0.006 * | |
| Indirect effects | | | |
| Lack of money | 0.1 (0.0, 0.1) | 0.006 * | 2.5 (0.6, 9.1) |
| Lack insurance | 0.0 (0.0, 0.1) | 0.31 | 1.2 (-2.0, 5.4) |
| Lack transportation | -3.5 (-10.2, 0.1) | 0.97 | 1.0 (-1927.876, 1812.497) |
| Lack of childcare | 0.0 (0.0, 0.1) | 0.93 | 0.0 (-0.9, 1.9) |
| Didn't know where to go for care | 0.1 (0.0, 0.2) | 0.1 | 2.8 (-0.9, 12.3) |
| Unable to find a provider | 0.0 (-0.1, 0.0) | 0.03 * | -0.9 (-3.5, -0.1) |
| Number of barriers | 0.2 (0.1, 0.3) | <0.001 * | 5.1 (2.7, 18.2) |
| | | | |
| All estimates adjusted by age and gender.<br>* Significant at the <0.05 level. | | | |
| | | | |
| a. Percent mediated = indirect effect/(direct effect + indirect effect) × 100 | | | |

© 2020 Brite J et al. *JAMA Network Open.*

eTable 10. Direct and indirect effect sizes of education on number of asthma ED visits among self-reported asthmatics

| **Education** | | | |
|---|---|---|---|
| | **Rate of ED visits per 100 enrollees** | | |
| At least a Bachelor's | 5.8 (5.0, 6.8) | | |
| Some college | 22.4 (19.9, 25.1) | | |
| High school | 17.5 (15.2, 20.1) | | |
| Less than high school | 29.9 (23.9, 37.5) | | |
| | **Change in number of ED visits per 100** | ***p-value*** | **% Mediated (95% CI) [a]** |
| Some college v. at least a Bachelor's | | | |
| Total effect | 18.7 (16.3, 21.2) | <0.001 * | |
| Indirect effects | | | |
| Lack of money | 0.6 (-0.1, 1.3) | 0.1 | 2.9 (-0.4, 6.3) |
| Lack insurance | 0.4 (-0.6, 1.4) | 0.44 | 1.7 (-2.9, 6.5) |
| Lack transportation | 1.0 (0.3, 1.8) | <0.001 * | 4.7 (1.8, 8.8) |
| Lack of childcare | 0.0 (-0.2, 0.0) | 0.23 | -0.2 ( -0.8, 0.2) |
| Didn't know where to go for care | 0.6 (0.1, 1.3) | 0.02 * | 3.1 (0.5, 6.4) |
| Unable to find a provider | 0.0 (-0.1, 0.0) | 0.47 | -0.1 (-0.5, 0.2) |
| Number of barriers | 0.7 (0.3, 1.0) | <0.001 * | 3.5 (1.7, 5.4) |
| High school v. at least a Bachelor's | | | |
| Total effect | 13.0 (10.4, 15.9) | <0.001 * | |
| Indirect effects | | | |
| Lack of money | 0.9 (0.2, 1.7) | 0.01 * | 6.2 (1.6, 11.6) |
| Lack insurance | 0.5 (-0.3, 1.6) | 0.23 | 3.6 (-2.4, 10.1) |
| Lack transportation | 0.7 (0.1, 1.4) | 0.018 * | 4.6 (1.0, 10.3) |
| Lack of childcare | -0.1 (-0.2, 0.0) | 0.13 | -0.4 (-1.5, 0.1) |
| Didn't know where to go for care | 0.3 (-0.2, 0.7) | 0.22 | 1.8 (-1.1, 5.2) |
| Unable to find a provider | 0.0 (-0.1, 0.0) | 0.30 | -0.2 (-0.9, 0.2) |
| Number of barriers | 0.6 (0.3, 1.0) | <0.001 * | 4.6 (2.3, 7.5) |
| Less than high school v. at least a Bachelor's | | | |
| Total effect | 25.7 (18.9, 33.9) | <0.001 * | |
| Indirect effects | | | |
| Lack of money | 2.7 (0.8, 5.7) | 0.004 * | 8.5 (3.1, 16.9) |
| Lack insurance | 3.3 (0.5, 7.3) | 0.002 * | 9.7 (1.9, 20.2) |
| Lack transportation | 2.0 (0.3, 5.0) | 0.004 * | 6.1 (1.1, 14.7) |
| Lack of childcare | -0.2 (0.0, 0.0) | 0.31 | -0.4 (-2.3, 0.1) |
| Didn't know where to go for care | 2.6 (0.9, 5.4) | <0.001 * | 8.4 (3.3, 16.6) |
| Unable to find a provider | -0.2 (-0.5, 0.1) | 0.21 | -0.5 (-1.8, 0.4) |
| Number of barriers | 2.4 (1.5, 3.8) | <0.001 * | 9.6 (6.3, 13.9) |
| | | | |
| All estimates adjusted by age and gender. | | | |
| * Significant at the <0.05 level. | | | |
| a. Percent mediated = indirect effect/(direct effect + indirect effect) × 100 | | | |

© 2020 Brite J et al. *JAMA Network Open.*
